# Association of Exposure to Particulate Matter Air Pollution With Semen Quality Among Men in China

**DOI:** 10.1001/jamanetworkopen.2021.48684

**Published:** 2022-02-17

**Authors:** Yan Zhao, Qianqian Zhu, Jiaying Lin, Jing Cai

**Affiliations:** 1 Shanghai Key Laboratory of Maternal Fetal Medicine, Shanghai First Maternity and Infant Hospital, School of Medicine, Tongji University, Shanghai, China; 2 Department of Assisted Reproduction, Shanghai Ninth People’s Hospital, Jiao Tong University School of Medicine, Shanghai, China; 3 School of Public Health, Key Lab of Public Health Safety of the Ministry of Education, NHC Key Laboratory of Health Technology Assessment, Fudan University, Shanghai, China

## Abstract

**Importance:**

The health effects of exposure to particulate matter (PM) on male fertility remain unclear. Although PM exposure has been linked with semen quality, the results were inconsistent.

**Objective:**

To examine the association of different size fractions of PM (<2.5 μm [PM_2.5_], 2.5-10 μm [PM_2.5-10_], and ≤10 μm [PM_10_]) exposure with semen quality in China.

**Design, Setting, and Participants:**

This retrospective cohort study was conducted using data on men whose wives underwent assisted reproductive technology procedures in a tertiary hospital in Shanghai, China, from January 1, 2013, to December 31, 2019. Participants were from 340 prefecture-level cities of China and were followed up from the date of their first visit until December 31, 2019. Data were analyzed from December 1, 2020, to May 15, 2021.

**Exposures:**

Exposure to PM_2.5_, PM_2.5-10_, and PM_10_ during the entire period (0-90 days before semen ejaculation) and key periods of sperm development (0-9, 10-14, and 70-90 days before semen ejaculation).

**Main Outcomes and Measures:**

Semen quality, including sperm count, concentration, and motility.

**Results:**

A total of 33 876 men were included in the final analysis, with a mean (SD) age of 34.1 (5.7) years and large variation of PM exposure. For example, the median exposure to PM_2.5_ during the entire period of sperm development was 46.05 (IQR, 34.38-61.65) μg/m^3^. During the entire period of sperm development, exposures to higher levels of PM were significantly associated with lower total and progressive sperm motility. For total sperm motility, an IQR increase in PM_2.5_ exposure was associated with an estimated effect decrease of −3.60% (95% CI, −3.93% to −3.26%); an increase in PM_2.5-10_ exposure, with an estimated effect decrease of −0.45% (95% CI, −0.76% to −0.14%); and an increase in PM_10_ exposure, with an estimated effect decrease of −2.44% (95% CI, −2.91% to −1.96%). Similar results were observed for progressive motility. An IQR increase in PM_2.5_ or PM_10_ exposures was associated with an estimated effect decrease on sperm progressive motility of −1.87% (95% CI, −2.37% to −1.36%) and −1.05% (95% CI, −1.45% to −0.64%), respectively. However, no significant associations were observed between PM exposure during the entire period of sperm development and sperm count or concentration.

**Conclusions and Relevance:**

These findings suggest that PM exposure may adversely affect sperm motility and highlight the need to reduce ambient particulate air pollution exposure for reproductive-aged men.

## Key Points

**Question:**

Is exposure to different fractions of particulate matter (<2.5, 2.5-10, and ≤10 μm in diameter) associated with poor semen quality?

**Findings:**

In this cohort study of 33 876 Chinese men, decreased total and progressive sperm motility and increased risk of asthenozoospermia were associated with exposure to particulate matter of less than 2.5 μm and 10.0 μm or less.

**Meaning:**

These findings suggest that control measures to reduce exposure to ambient particulate matter may help increase male fertility and reduce the risk of asthenozoospermia.

## Teaser

This cohort study evaluates the association between exposure to different particulate matter fractions and poor semen quality among Chinese men whose wives underwent assisted reproductive technology procedures.

## Introduction

Infertility is becoming a global public health issue, affecting approximately 10% of all reproductive-aged couples worldwide.^1,2^ The World Health Organization estimates that pure male factors, mainly poor semen quality, could account for 50% of infertility cases.^3^ In recent years, extensive evidence has suggested a global downward trend in semen quality, and multiple studies have reported significant declines in sperm concentration, count, and motility in recent decades.^4,5,6,7^

Both genetic background and environmental factors contribute to poor semen quality.^8,9,10,11^ The remarkable changes in sperm concentration, count, and motility over a relatively short period suggest that the global downward trend in semen quality is more likely to be related to environmental factors than genetics.^12,13^

Particulate matter (PM) air pollution is a serious environmental threat worldwide.^14,15^ Recent human epidemiological studies have reported an association between PM exposure and semen quality.^16,17^ However, findings from these studies have been inconsistent, with some,^18,19,20,21^ but not all,^22,23,24^ studies reporting negative associations. In addition, previous studies have focused primarily on PM with aerodynamic diameters of 10 μm or less (PM_10_) or less than 2.5 μm (PM_2.5_). The potential effects of PM with aerodynamic diameters of 2.5 to 10 μm (PM_2.5-10_) remain under investigation. Moreover, most previous studies were conducted in single regions with relatively small sample sizes, which may limit the generalizability of their findings.

Considering the severe particulate air pollution^25^ and accelerating decline in semen quality in China,^4,7^ national studies are needed to assess the association between PM exposure and semen quality. Therefore, we conducted this nationwide study among 33 876 men from 340 Chinese cities to evaluate the association between exposure to different PM size fractions (PM_2.5_, PM_2.5-10_, and PM_10_) and poor semen quality.

## Methods

### Study Population

This retrospective cohort study consisted of male patients who visited the Reproductive Medical Center of Shanghai Ninth People’s Hospital in Shanghai, China, and whose wives were seeking assisted reproductive technology procedures from January 1, 2013, to December 31, 2019. Men were excluded once any of the following conditions were met: chromosomal abnormality, varicocele, azoospermia, difficult sperm retrieval, reproductive tract infection, genital tract trauma, history of mumps, and vasectomy surgery.

The included participants were interviewed, and data on detailed residential address, age, ethnicity, body weight and height, educational level, cigarette smoking, and alcohol consumption were collected by trained physicians. Because all data were used anonymously, written consent was not obtained from each participant. Ethics approval was obtained from the Ethics Committee of Shanghai Ninth People’s Hospital. This study followed the Strengthening the Reporting of Observational Studies in Epidemiology (STROBE) reporting guideline.

### Semen Analysis

After their number of abstinence days were recorded, the participant’s semen samples were collected via ejaculation and liquefied in a 37 °C heating chamber for no more than 60 minutes. The semen parameters of sperm count, sperm concentration, and sperm motility (total and progressive) were evaluated using a computer-assisted semen analysis system (SSA-II; Beijing Suijia Software Co, Ltd) according to the World Health Organization guidelines for the examination of human semen.^26^ The detailed descriptions of the computer-assisted semen analysis are provided in eMethods of the Supplement.

Sperm motility parameters included progressive and total motility. Motility was graded as follows: A indicated rapid progressive motility; B, slow progressive motility; C, local motility; and D, immotility. Progressive motility was defined as the proportion of rapid and slow-progressive spermatozoa. Nonprogressive motility was defined as the proportion of spermatozoa with local motility. Total motility was calculated by summing progressive and nonprogressive motilities. Quality control procedures were established by the semen laboratory according to World Health Organization (2010) guidelines^26^ and were routinely conducted by laboratory technicians. All tests were in agreement with the quality control standards.

### Exposure Assessment

Daily (24-hour) particulate air pollution data, including PM_2.5_ and PM_10_, in the nearest monitoring station of each participant were obtained from the National Urban Air Quality Real-Time Publishing Platform, which is administered by the China National Environment Monitoring Center. Routine measurement of PM_2.5_ and PM_10_ at monitoring stations was performed using the tapered element oscillating microbalance method under the China National Quality Control. Daily PM_2.5-10_ levels at each station were calculated by subtracting the daily PM_2.5_ from the daily PM_10_. To allow the adjustment for concomitant exposure to gaseous pollutants, we also obtained daily concentrations of nitrogen dioxide, sulfur dioxide, carbon monoxide, and ozone from the same monitoring stations. Moreover, to adjust for the potential confounding effect of weather conditions, we collected daily mean temperature and relative humidity from the China Meteorological Data Sharing Service System.

Exposures to PM_2.5_, PM_2.5-10_, and PM_10_ were estimated based on the daily concentrations of the nearest monitoring stations. We estimated individual exposures to PM_2.5_, PM_2.5-10_, and PM_10_ during the entire period of sperm development (0-90 days before semen ejaculation) and the 3 key periods of sperm development, including epididymal storage (0-9 days before semen ejaculation), sperm motility development (10-14 days before semen ejaculation), and spermatogenesis (70-90 days before semen ejaculation) for each participant.

### Statistical Analysis

Data were analyzed from December 1, 2020, to May 15, 2021. Given that sperm count and concentration are right skewed, these 2 parameters were natural logarithm transformed before fitting in statistical models to improve the approximation of the normal distribution. We first examined the associations among PM_2.5_, PM_2.5-10_, and PM_10_ exposures during the entire period of sperm development and measures of semen parameters using linear mixed-effect (LME) models with each participant’s province of residence as the random intercept. The aforementioned exposure measures were in turn fitted into LME models as independent variables, whereas semen parameters were fitted as dependent variables. We selected the following potential confounders according to their associations with PM exposure or semen quality: ethnicity (Han or other), age (<30, 31-39, and ≥40 years), body mass index (calculated as weight in kilograms divided by height in meters squared [<18.5, 18.5-23.9, or ≥24.0]), educational level (middle school and lower, high school, or college and higher), current smoking (yes or no), alcohol consumption (yes or no), season of semen collection (spring, summer, autumn, or winter), temperature, relative humidity, and gaseous pollutants.^27,28,29^ We estimated the effect estimates and 95% CIs for each semen quality parameter in association with an IQR increase in PM exposure. We then examined the exposure-response associations by grouping PM exposure levels into quartiles (as independent variable) and estimated the regression coefficients with the first quartile as the reference.

Regarding the association between PM exposure during the 3 key periods of sperm development (spermatogenesis, sperm motility development, and epididymal storage) and measures of sperm motility, we conducted multivariate LME models in which progressive or total motility was fitted as the dependent variable and continuous PM exposure data during each key period were entered as the independent variable. Multivariate LME models were adjusted for the aforementioned confounders.

Given that previous studies have reported that smoking and drinking are known risk factors for poor sperm motility,^30,31^ we conducted a comparison to see whether those 2 confounders play a role in the association of PM exposure with sperm motility by including and excluding them in the LME model. To explore the potential effect modifiers, we further conducted stratified analyses by age, body mass index, and educational level.

Finally, we conducted logistic regression analysis using generalized additive mixed models to estimate the associations between PM exposure during the entire period of sperm development (as independent variable) and the risk of asthenozoospermia (a dichotomous outcome and defined as progressive motility <32%). We fitted a random contribution of province and adjusted for the same confounders as in LME models. The odds ratios and 95% CIs for each IQR increase in PM exposures during the entire period of sperm development were calculated. All analyses were performed using R, version 3.2.3 (R Project for Statistical Computing). All tests were 2 sided, and *P* < .05 was considered statistically significant.

## Results

### Participant Characteristics

A total of 33 876 men were included in the final analysis, with a mean (SD) age of 34.1 (5.7) years; 16 725 of the men (49.4%) were overweight or obese, and 33 560 (99.1%) were of Chinese Han ethnicity (Table 1). Nine thousand seven hundred thirty-eight men (28.7%) were current smokers, whereas only 456 (1.3%) reported current alcohol consumption. The median sperm count was 168.9 (IQR, 87.0-279.0) × 10^6^ and the median sperm concentration was 69.0 (IQR, 37.3-104.5) × 10^6^/mL. The mean (SD) total and progressive motility were 55.5% (23.4%) and 43.5% (19.7%), respectively. Overall decreased total (52.2% [22.7%]) and progressive (41.5% [19.2%]) sperm motility were observed for high-level exposure to PM_2.5_.

**Table 1.** Characteristics and Semen Parameters of the Participating Men by PM_2.5_ Exposure During the Entire Period of Sperm Development^a^

Characteristic	Overall (N = 33 876)	Quartile of PM_2.5_ exposure			
		1st (n = 8474)	2nd (n = 8466)	3rd (n = 8474)	4th (n = 8462)
Ethnicity					
Han	33 560 (99.1)	8400 (99.1)	8378 (99.0)	8408 (99.2)	8374 (99.0)
Other	316 (0.9)	74 (0.9)	88 (1.0)	66 (0.8)	88 (1.0)
Age, y					
<30	8558 (25.3)	1997 (23.6)	2060 (24.3)	2151 (25.4)	2350 (27.8)
31-39	20 283 (59.9)	5114 (60.3)	5169 (61.1)	5063 (59.7)	4937 (58.3)
≥40	5035 (14.9)	1363 (16.1)	1237 (14.6)	1260 (14.9)	1175 (13.9)
BMI					
<18.5	973 (2.9)	274 (3.2)	213 (2.5)	242 (2.9)	244 (2.9)
18.5-23.9	16 178 (47.8)	4115 (48.6)	4075 (48.1)	3961 (46.7)	4027 (47.6)
≥24.0	16 725 (49.4)	4085 (48.2)	4178 (49.4)	4271 (50.4)	4191 (49.5)
Educational level					
Middle school or less	4586 (13.5)	1313 (15.5)	1028 (12.1)	1025 (12.1)	1220 (14.4)
High school	11 967 (35.3)	2862 (33.8)	2856 (33.7)	3086 (36.4)	3163 (37.4)
College or above	17 323 (51.1)	4299 (50.7)	4582 (54.1)	4363 (51.5)	4079 (48.2)
Cigarette smoking	9738 (28.7)	2690 (31.7)	2489 (29.4)	2342 (27.6)	2217 (26.2)
Alcohol consumption	456 (1.3)	112 (1.3)	124 (1.5)	115 (1.4)	105 (1.2)
Season of semen collection					
Spring	10 322 (30.5)	694 (8.2)	1495 (17.7)	3660 (43.2)	4473 (52.9)
Summer	8539 (25.2)	2800 (33.0)	2962 (35.0)	2296 (27.1)	481 (5.7)
Autumn	8701 (25.7)	4548 (53.7)	3040 (35.9)	916 (10.8)	197 (2.3)
Winter	6314 (18.6)	432 (5.1)	969 (11.4)	1602 (18.9)	3311 (39.1)
Semen parameters					
Count, median (IQR), ×10^6^	168.9 (87.0-279.0)	169.7 (91.5-269.6)	166.4 (85.0-277.5)	167.5 (84.3-282.2)	171.8 (86.8-285.9)
Concentration, median (IQR), ×10^6^/mL	69.0 (37.3-104.5)	68.6 (38.6-100.2)	69.3 (36.8-105.1)	68.8 (37.0-107.6)	69.4 (36.5-106.8)
Motility, mean (SD), %					
Total	55.5 (23.4)	59.6 (22.8)	55.8 (23.8)	54.6 (23.7)	52.2 (22.7)
Progressive	43.5 (19.7)	46.0 (19.3)	43.5 (20.0)	42.9 (20.0)	41.5 (19.2)

Abbreviations: BMI, body mass index (calculated as weight in kilograms divided by height in meters squared); PM_2.5_, particulate matter with aerodynamic diameter of less than 2.5 μm.

^a^ Indicates 0 to 90 days before semen ejaculation. Unless otherwise indicated, data are expressed as number (%) of patients. Percentages have been rounded and may not total 100.

### PM Exposure Levels

As shown in eFigure 1 in the Supplement, the 33 876 participants resided across 340 prefecture-level cities in 31 provinces of China. The median levels of PM_2.5_, PM_2.5-10_, and PM_10_ exposure during the entire period of sperm development were 46.05 (IQR, 34.38-61.65) μg/m^3^, 25.09 (IQR, 19.31-34.15) μg/m^3^, and 72.43 (IQR, 56.63-93.17) μg/m^3^, respectively (eTable 1 in the Supplement). As shown in eTable 2 in the Supplement, the mean PM_2.5_, PM_2.5-10_, and PM_10_ exposure levels during the entire period of sperm development generally peaked in 2014 and then exhibited a decreasing trend afterward. eFigures 2, 3, and 4 in the Supplement show the distribution of PM_2.5_, PM_2.5-10_, and PM_10_ exposure, respectively, during the entire period of sperm development for each prefecture-level city. We found that participants living in the middle and lower reaches of the Yellow River, the Jing-Jin-Ji region, and Xinjiang consistently experienced higher PM exposure (eg, PM_2.5_ >55.8 μg/m^3^), compared with other regions of China.

The correlations between PM and gaseous pollutant exposure during the entire period of sperm development are presented in eTable 3 in the Supplement. The results showed that PM_2.5_ exposure levels were highly correlated with PM_10_ exposure levels (Spearman *r* = 0.89) and weakly correlated with PM_2.5-10_ exposure levels (Spearman *r* = 0.44). Regarding gaseous pollutants and meteorological parameters, we found weak to moderate correlations with PM (Spearman *r* range, −0.67 to 0.65).

### Associations of PM Exposure During the Entire Period of Sperm Development With Semen Quality

Table 2 presents the associations between PM exposure during the entire period of sperm development and semen quality measures. Exposures to PM_2.5_, PM_2.5-10_, and PM_10_ were not associated with sperm motility. In the adjusted model, an IQR increase in PM_2.5_ exposure was associated with an effect estimate of reduced total motility of −3.60% (95% CI, −3.93% to −3.26%), an increase in PM_2.5-10_ exposure of −0.45% (95% CI, −0.76% to −0.14%), and an increase in PM_10_ exposure of −2.44% (95% CI, −2.91% to −1.96%). Similar results were observed for progressive motility in which an IQR increase in PM_2.5_ and PM_10_ exposure was associated with a reduction in estimated effect of −1.87% (95% CI, −2.37% to −1.36%) and −1.05% (95% CI, −1.45% to −0.64%). However, no significant associations were observed between PM exposure and sperm count or concentration.

**Table 2.** Effect Estimates of Semen Parameters Associated With PM Exposure During the Entire Period of Sperm Development^a^

Semen parameters	Model, effect estimate (95% CI), %	
	Crude	Adjusted^b^
Count		
PM_2.5_	−0.08 (−1.45 to 1.29)	1.33 (−1.00 to 3.67)
PM_2.5-10_	0.85 (−0.21 to 1.91)	0.47 (−0.70 to 1.65)
PM_10_	−0.09 (−1.36 to 1.18)	0.08 (−1.72 to 1.89)
Concentration		
PM_2.5_	0.47 (−0.83 to 1.76)	1.31 (−0.95 to 3.57)
PM_2.5-10_	0.55 (−0.49 to 1.58)	0.19 (−1.00 to 1.37)
PM_10_	0.06 (−1.16 to 1.29)	−0.26 (−2.07 to 1.56)
Total motility		
PM_2.5_	−4.27 (−4.86 to −3.67)^c^	−3.60 (−3.93 to −3.26)^c^
PM_2.5-10_	−1.30 (−1.58 to −1.02)^c^	−0.45 (−0.76 to −0.14)^c^
PM_10_	−3.07 (−3.40 to −2.75)^c^	−2.44 (−2.91 to −1.96)^c^
Progressive motility		
PM_2.5_	−2.18 (−2.46 to −1.89)^c^	−1.87 (−2.37 to −1.36)^c^
PM_2.5-10_	−0.74 (−0.97 to −0.50)^c^	−0.13 (−0.39 to 0.14)
PM_10_	−1.85 (−2.12 to −1.57)^c^	−1.05 (−1.45 to −0.64)^c^

Abbreviations: PM_2.5_, particulate matter with aerodynamic diameter of less than 2.5 μm; PM_2.5-10_, PM with aerodynamic diameter of 2.5 to 10 μm; PM_10_, PM with aerodynamic diameter of 10 μm or less.

^a^ Indicates 0 to 90 days before semen ejaculation. For sperm count and concentration, effect estimates represented their percentage changes in association with an IQR increase in PM exposure. For total and progressive motility, effect estimates represented their absolute changes in association with an IQR increase in PM exposure.

^b^ Adjusted for ethnicity, age, educational level, body mass index, smoking, alcohol consumption, season of semen collection, abstinence period, temperature, relative humidity, and gaseous pollutants.

^c^ Two-sided *P* < .01.

We then examined the exposure-response associations of PM exposure with sperm motility by categorizing the distribution of PM exposure levels into quartiles. As shown in the Figure, dose-response associations were observed between increasing PM_2.5_ or PM_10_ exposure levels and decreasing sperm motility, as suggested by the monotonic decrease in sperm total and progressive motility across quartiles of PM_2.5_ or PM_10_ exposure levels. However, no exposure-response associations were observed between PM_2.5-10_ exposure and sperm motility.

**Figure.**
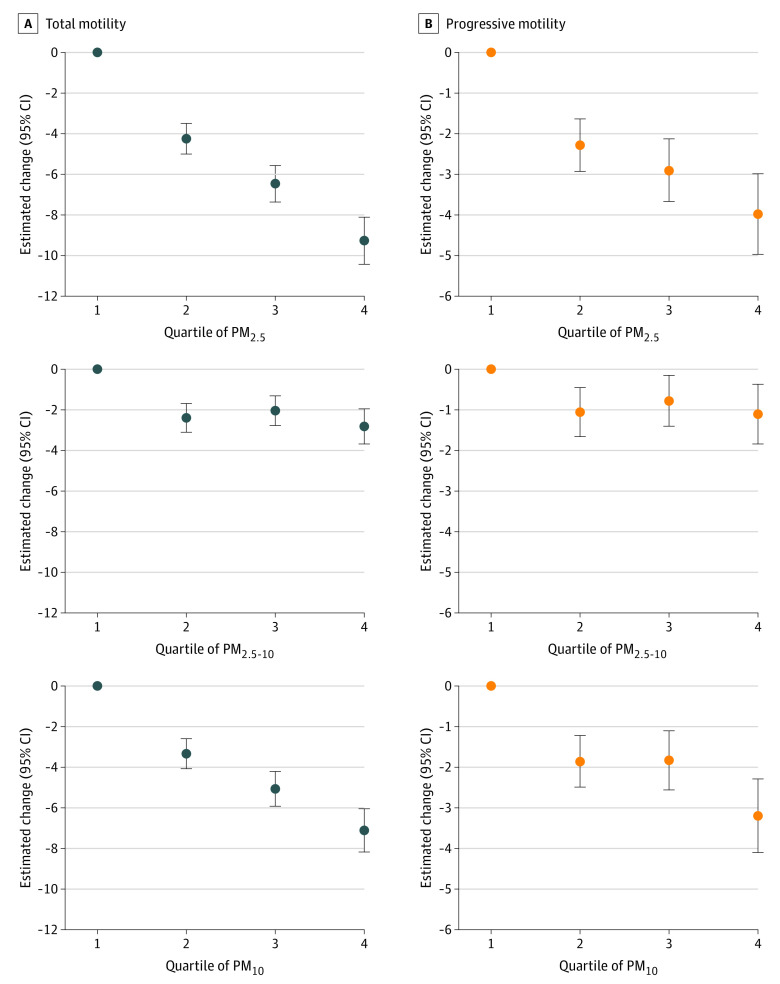
Estimated Changes in Total and Progressive Sperm Motility Associated With Quartile of Particulate Matter (PM) Exposure During the Entire Period of Sperm Development Error bars indicate 95% CIs. Particulate matter exposure is measured as aerodynamic diameters of less than 2.5 μm (PM_2.5_), 2.5 to 10 μm (PM_2.5-10_), and 10 μm or less (PM_10_). All models are adjusted for ethnicity, age, educational level, body mass index, smoking, alcohol consumption, season of semen collection, abstinence period, temperature, relative humidity, and gaseous pollutants.

### Critical Exposure Windows for the Associations of PM Exposure With Semen Motility

Based on our observations of the adverse effects of PM exposure on sperm motility, we further explored the possible critical exposure windows for these adverse effects. We calculated the effect estimates for the 3 key periods of sperm development (ie, spermatogenesis, sperm motility development, and epididymal storage). As shown in Table 3, exposures to PM for those 3 key periods of sperm development were all negatively associated with sperm motility. The effect estimates of PM_2.5_ and PM_10_ for the period of spermatogenesis were significantly larger than the periods of sperm motility development and epididymal storage, because the 95% CIs of the former and the latter did not overlap. For example, the effect estimates of PM_2.5_ on total motility were −3.27% (95% CI, −3.76% to −2.79%) for the period of spermatogenesis, −1.42% (95% CI, −1.80% to −1.04%) for the period of sperm motility development, and −1.06% (95% CI, −1.48% to −0.64%) for the period of epididymal storage.

**Table 3.** Effect Estimates of Semen Motility Parameters Associated With PM Exposure During Specific Time Windows of Sperm Development

Exposure time window, lag days^b^	Motility, effect estimate (95% CI), %^a^	
	Total	Progressive
0-9		
PM_2.5_	−1.06 (−1.48 to −0.64)^c^	−0.27 (−0.63 to 0.09)
PM_2.5-10_	−0.34 (−0.63 to −0.05)^d^	−0.04 (−0.29 to 0.21)
PM_10_	−0.91 (−1.33 to −0.50)^c^	−0.16 (−0.52 to 0.19)
10-14		
PM_2.5_	−1.42 (−1.80 to −1.04)^c^	−0.63 (−0.95 to −0.30)^c^
PM_2.5-10_	−0.17 (−0.46 to 0.11)	0.04 (−0.20 to 0.28)
PM_10_	−1.02 (−1.40 to −0.63)^c^	−0.33 (−0.66 to −0.01)^d^
70-90		
PM_2.5_	−3.27 (−3.76 to −2.79)^c^	−1.62 (−2.03 to −1.21)^c^
PM_2.5-10_	−0.40 (−0.71 to −0.09)^d^	−0.15 (−0.41 to 0.11)
PM_10_	−2.21 (−2.67 to −1.75)^c^	−0.99 (−1.38 to −0.60)^c^

Abbreviations: PM_2.5_, particulate matter with aerodynamic diameter of less than 2.5 μm; PM_2.5-10_, PM with aerodynamic diameter of 2.5 to 10 μm; PM_10_, PM with aerodynamic diameter of 10 μm or less.

^a^ Models were adjusted for ethnicity, age, educational level, body mass index, smoking, alcohol consumption, season of semen collection, abstinence period, temperature, relative humidity, and gaseous pollutants. Effect estimates represent the absolute changes of sperm total motility and progressive motility in association with an IQR increase in PM exposure.

^b^ Zero to 9 lag days indicates the period of epididymal storage; 10 to 14 lag days, the period of sperm motility development; and 70 to 90 lag days, the periods of spermatogenesis.

^c^ Two-sided *P* < .01.

^d^ Two-sided *P* < .05.

### Sensitivity Analyses and Stratified Analyses

The results of the sensitivity analyses are presented in eTable 4 in the Supplement. Results of the unadjusted analysis (smoking and alcohol consumption) were generally similar to those of adjusted analysis, although effect estimates became relatively larger. In stratified analyses, we found stronger associations between PM exposure and total (eTable 5 in the Supplement) or progressive (eTable 6 in the Supplement) motility among men with higher educational levels or older than 40 years. However, these differences in estimated effects were generally nonsignificant (overlapping 95% CIs).

### Associations of PM Exposure During the Entire Period of Sperm Development With Risk of Asthenozoospermia

The associations between PM exposure during the entire period of sperm development and the risk of asthenozoospermia are shown in Table 4. We observed increased odds of asthenozoospermia in association with elevated PM_2.5_ or PM_10_ exposures during the entire period of sperm development. In an adjusted model, per IQR increase in PM_2.5_ or PM_10_ exposure was associated with a 14% (odds ratio, 1.14 [95% CI, 1.07-1.20]) and a 9% (odds ratio, 1.09 [95% CI, 1.04-1.14]) increase in the odds of asthenozoospermia. However, no significant associations were observed between PM_2.5-10_ exposure and the risk of asthenozoospermia.

**Table 4.** Odds of Asthenozoospermia Associated With PM Exposure During the Entire Period of Sperm Development^a^

Pollutant	Model, OR (95% CI)	
	Crude	Adjusted^b^
PM_2.5_	1.20 (1.17-1.24)^c^	1.14 (1.07-1.20)^c^
PM_2.5-10_	1.07 (1.04-1.10)^d^	1.01 (0.98-1.04)
PM_10_	1.18 (1.14-1.21)^c^	1.09 (1.04-1.14)^d^

Abbreviations: OR, odds ratio; PM_2.5_, particulate matter with aerodynamic diameter of less than 2.5 μm; PM_2.5-10_, PM with aerodynamic diameter of 2.5 to 10 μm; PM_10_, PM with aerodynamic diameter of 10 μm or less.

^a^ Includes 0 to 90 days before semen ejaculation.

^b^ Adjusted for ethnicity, age, educational level, body mass index, smoking, alcohol consumption, season of semen collection, abstinence period, temperature, relative humidity, and gaseous pollutants.

^c^ Two-sided *P* < .01.

^d^ Two-sided *P* < .05.

## Discussion

This study examined the potential associations of PM_2.5_, PM_2.5-10_, and PM_10_ exposure with semen quality among 33 876 men from 340 cities in China. We found that PM exposures during the entire period of sperm development were inversely associated with sperm total motility and progressive motility but not sperm count and concentration. Moreover, we found a significantly increased risk of asthenozoospermia in association with elevated PM_2.5_ or PM_10_ exposure levels. To our knowledge, this is the first nationwide study to examine the association between PM exposure and semen quality in China.

Previous studies^32,33,34^ have also examined the associations between PM exposure during the entire period of sperm development and sperm motility. Consistent with our findings, Qiu et al^32^ reported that the 90-day mean concentrations of PM_2.5_ and PM_10_ were negatively correlated with forward motility in their analysis of longitudinal data from a human sperm bank. Guan et al^33^ also observed a significant inverse association between PM_10_ exposure during the entire period of spermatogenesis and sperm total motility in 1955 men and 2073 semen samples. Similarly, in a study including 1061 men attending an infertility clinic in Wuhan, China, Sun et al^34^ observed that PM_10_ exposure during the entire period of spermatogenesis was negatively associated with both total and progressive sperm motility. However, inconsistent results have also been reported. Two studies conducted in the province of Wuhan^18,20^ and 1 study in Guangdong^35^ did not observe any association between PM exposure and sperm motility. These inconsistent findings might be due to the relatively small sample sizes of the studies, which may not have been able to detect the subtle effects of PM exposure on sperm motility.

In addition to the significant negative associations between PM exposure during the entire period of sperm development and sperm motility, we also explored the possible critical exposure window for the adverse effects of PM exposure on semen motility. We found the effect estimates during the period of spermatogenesis were significantly larger than the other 2 windows (the periods of sperm motility development and epididymal storage), which suggest that PM exposure during the period of spermatogenesis may play a more important role on semen motility than the other 2 exposure windows. Previous evidence has shown that PM could disrupt the synthesis of proteins necessary for sperm motility.^35^ We speculated that PM exposure during this key period of sperm development may alter the expression of genes involved in protein transcription and translation.

Different size fractions of PM have been proposed to have differing effects on semen quality. However, few studies have investigated the relative importance of different size fractions of PM to induce such an effect. We found evidence of an adverse association of PM_2.5_, PM_2.5-10_, and PM_10_ with sperm motility. However, the effect estimates for PM_10_ were weaker than those for PM_2.5_ and stronger than those for PM_2.5-10_. Because more than 50% of the PM_10_ mass consists of PM_2.5_,^36^ we hypothesized that the effects of PM_10_ on sperm motility were primarily driven by PM_2.5_. Our findings suggest that smaller PM size fractions may be more potent than larger fractions in inducing poor sperm motility.

The biological mechanisms by which PM exposure may impair sperm motility development have yet to be determined. Both PM_2.5_ and PM_10_ exposure can induce excess reactive oxygen species in humans.^37,38^ The overproduction of reactive oxygen species can damage the blood-testis barrier and negatively affect spermatogenesis, leading to decreased sperm motility.^39,40,41^ In addition, PM exposures cause systemic inflammation by increasing tumor necrosis factor and interleukin 1β levels.^42,43,44^ Higher tumor necrosis factor and interleukin 1β levels are associated with reduced sperm motility.^45,46,47^ We propose that increased oxidative stress and inflammatory reactions induced by PM_2.5_ or PM_10_ may partly account for the decline in sperm motility. Further toxicological studies are required to clarify the detailed mechanisms underlying the PM-induced decline in sperm motility.

Poor sperm motility has raised global concern as a major cause of male infertility. Our findings add evidence that PM exposure during sperm motility development may contribute to reduced sperm motility. Although the estimated decrease in sperm motility was relatively small, it still resulted in significantly increased odds of asthenozoospermia. Considering the downward trend in sperm motility^4^ and severe particulate air pollution in China,^25^ our findings may have important public health implications.

### Limitations

This study has some limitations. First, we used monitoring data from fixed stations rather than personal measurements as a proxy measure for PM exposure. Thus, exposure misclassification may be inevitable. Second, although our analyses adjusted for a variety of confounders, unmeasured confounders such as dietary habits, physical condition, and exposure to other environmental pollutants were possible.^48,49,50^ Third, owing to a lack of data, we were unable to investigate the effects of PM exposure on sperm morphology.

## Conclusions

Our findings suggest that exposure to particulate air pollution during spermatogenesis may adversely affect semen quality, especially sperm motility, and highlight the need to reduce ambient particulate air pollution exposure in reproductive-aged men. Further studies are needed to determine the biological mechanisms underlying the observed associations.

## Notes

Supplement. **eMethods.** Computer-Assisted Semen Analysis of the Semen Parameters **eTable 1.** Distribution of PM Exposure During the Entire Period and Key Period of Sperm Development (μg/m^3^) **eTable 2.** Mean PM_2.5_, PM_2.5-10_, and PM_10_ Exposure During the Entire Period of Sperm Development (0-90 Days Before Semen Ejaculation) From 2013 to 2019 **eTable 3.** Spearman Correlation Coefficients Between PMs and Gaseous Pollutants During the Entire Period of Sperm Development (0-90 Days Before Semen Ejaculation) **eTable 4.** Effect Estimates and 95% CIs of Semen Total and Progressive Motility Associated With an IQR Increase in PM Exposure During the Entire Period of Sperm Development (0-90 Days Before Semen Ejaculation) With and Without Adjustment for Smoking and Alcohol Consumption **eTable 5.** Effect Estimates and 95% CIs of Total Motility Associated With an IQR Increase in PM Exposure During the Entire Period of Sperm Development (0-90 Days Before Semen Ejaculation) in Each Subgroup **eTable 6.** Effect Estimates and 95% CIs of Progressive Motility Associated With an IQR Increase in PM Exposure During the Entire Period of Sperm Development (0-90 Days Before Semen Ejaculation) in Each Subgroup **eFigure 1.** Location of Participants’ Residence Cities **eFigure 2.** Distribution of Exposure to PM_2.5_ During the Entire Period Of Sperm Development for Participating Men Residing in Prefecture-Level Cities **eFigure 3.** Distribution of Exposure to PM_2.5-10_ During the Entire Period of Sperm Development for Participating Men Residing in Prefecture-Level Cities **eFigure 4.** Distribution of Exposure to PM_10_ During the Entire Period of Sperm Development for Participating Men Residing in Prefecture-Level Cities
