# Supplementary material for: Association of Exposure to Particulate Matter Air Pollution With Semen Quality Among Men in China
Source: JAMA Netw Open. 2022 Feb 17;5(2):e2148684. doi: 10.1001/jamanetworkopen.2021.48684 (PMC8855237; doi:10.1001/jamanetworkopen.2021.48684)
Supplement: Supplement. — eMethods. Computer-Assisted Semen Analysis of the Semen Parameters eTable 1. Distribution of PM Exposure During the Entire Period and Key Period of Sperm Development (μg/m3) eTable 2. Mean PM2.5, PM2.5-10, and PM10 Exposure During the Entire Period of Sperm Development (0-90 Days Before Semen Ejaculation) From 2013 to 2019 eTable 3. Spearman Correlation Coefficients Between PMs and Gaseous Pollutants During the Entire Period of Sperm Development (0-90 Days Before Semen Ejaculation) eTable 4. Effect Estimates and 95% CIs of Semen Total and Progressive Motility Associated With an IQR Increase in PM Exposure During the Entire Period of Sperm Development (0-90 Days Before Semen Ejaculation) With and Without Adjustment for Smoking and Alcohol Consumption eTable 5. Effect Estimates and 95% CIs of Total Motility Associated With an IQR Increase in PM Exposure During the Entire Period of Sperm Development (0-90 Days Before Semen Ejaculation) in Each Subgroup eTable 6. Effect Estimates and 95% CIs of Progressive Motility Associated With an IQR Increase in PM Exposure During the Entire Period of Sperm Development (0-90 Days Before Semen Ejaculation) in Each Subgroup eFigure 1. Location of Participants’ Residence Cities eFigure 2. Distribution of Exposure to PM2.5 During the Entire Period Of Sperm Development for Participating Men Residing in Prefecture-Level Cities eFigure 3. Distribution of Exposure to PM2.5-10 During the Entire Period of Sperm Development for Participating Men Residing in Prefecture-Level Cities eFigure 4. Distribution of Exposure to PM10 During the Entire Period of Sperm Development for Participating Men Residing in Prefecture-Level Cities [file jamanetwopen-e2148684-s001.pdf]

Supplementary Online Content

Zhao Y, Zhu Q, Lin J, Cai J. Association of exposure to particulate matter air pollution with semen quality among men in China. *JAMA Netw Open*. 2022;5(2):e2148684. doi:10.1001/jamanetworkopen.2021.48684

eMethods. Computer-Assisted Semen Analysis of the Semen Parameters
eTable 1. Distribution of PM Exposure During the Entire Period and Key Period of Sperm Development (μg/m^3)
eTable 2. Mean $PM_{2.5}$, $PM_{2.5\text{-}10}$, and PM_{10} Exposure During the Entire Period of Sperm Development (0-90 Days Before Semen Ejaculation) From 2013 to 2019
eTable 3. Spearman Correlation Coefficients Between PMs and Gaseous Pollutants During the Entire Period of Sperm Development (0-90 Days Before Semen Ejaculation)
eTable 4. Effect Estimates and 95% CIs of Semen Total and Progressive Motility Associated With an IQR Increase in PM Exposure During the Entire Period of Sperm Development (0-90 Days Before Semen Ejaculation) With and Without Adjustment for Smoking and Alcohol Consumption
eTable 5. Effect Estimates and 95% CIs of Total Motility Associated With an IQR Increase in PM Exposure During the Entire Period of Sperm Development (0-90 Days Before Semen Ejaculation) in Each Subgroup
eTable 6. Effect Estimates and 95% CIs of Progressive Motility Associated With an IQR Increase in PM Exposure During the Entire Period of Sperm Development (0-90 Days Before Semen Ejaculation) in Each Subgroup
eFigure 1. Location of Participants' Residence Cities
eFigure 2. Distribution of Exposure to $PM_{2.5}$ During the Entire Period Of Sperm Development for Participating Men Residing in Prefecture-Level Cities
eFigure 3. Distribution of Exposure to $PM_{2.5\text{-}10}$ During the Entire Period of Sperm Development for Participating Men Residing in Prefecture-Level Cities
eFigure 4. Distribution of Exposure to PM_{10} During the Entire Period of Sperm Development for Participating Men Residing in Prefecture-Level Cities

This supplementary material has been provided by the authors to give readers additional information about their work.

© 2022 Zhao Y et al. *JAMA Network Open*.

eMethods. Computer-Assisted Semen Analysis of the Semen Parameters

We followed World Health Organization (WHO) guidelines for samples collection and parameter analyzes of human semen.

1. Sample collection and preparation for computer-aided sperm analysis (CASA)

To limit the exposure of the semen to fluctuations in temperature and to control the time between collection and analysis, semen sample collection was conducted in a private room near the laboratory. Samples were collected after a minimum of 2 days and a maximum of 7 days of sexual abstinence. After their number of abstinence days were recorded, the participant's semen samples were collected via masturbation and liquefied in a 37°C heating chamber for no more than 60 min. We required our participants to obtain their semen samples by masturbation and used a clean, wide-mouthed container made of glass or plastic, from a batch that has been confirmed to be non-toxic for spermatozoa. All the specimen containers soon afterwards were kept at ambient temperature (i.e. between 20 °C and 37 °C), to avoid large changes in temperature that may affect the spermatozoa after they are ejaculated into it. The semen samples were then liquefied in a 37°C heating chamber for no more than 60 min.

2. Use of CASA to assess sperm motility

We used computer-assisted semen analysis (CASA) (SSA-II, Beijing Suijia Software, Beijing, China) to analyze the semen parameters of sperm count, sperm concentration, and sperm motility (i.e. total and progressive motility). The tracks of at least 200 motile spermatozoa per specimen were analyzed. During analysis, the CASA system maintained the specimen at 37 °C since sperm motion is sensitive to temperature. For

© 2022 Zhao Y et al. *JAMA Network Open*.

samples with high sperm concentrations (i.e., greater than 50×10^6 per ml), they were diluted with seminal plasma from the same man to avoid collisions.

A dual chamber system with disposable counting chambers were used to examine the semen parameters so that both chambers were filled with sample and assessed. To ensure the representativeness of examined fields six fields per chamber (12 fields in total) were read and in each chamber, we assessed 200 spermatozoa at least. The same principles of quality control were applied for standard estimations of motility.

3. Use of CASA to estimate sperm concentration

We used fluorescent DNA stains with CASA to determine the concentration of motile sperm and percentage motility. We assessed the sample at several different distances from the site of loading the chamber. A haemocytometer was also used for validation against. For some samples with high sperm concentrations (i.e., greater than 50×10^6 per ml), they were diluted before examination.

© 2022 Zhao Y et al. *JAMA Network Open*.

eTable 1. Distribution of PM Exposure During the Entire Period and Key Period of Sperm Development (μg/m^3)

Timing of measurement	Mean	SD	Percentile			IQR
			25th	50th	75th	
$PM_{2.5}$						
0-90 lag days	49.88	21.07	34.38	46.05	61.65	27.27
0-9 lag days	47.85	25.92	30.46	43.11	58.57	28.11
10-14 lag days	48.66	30.67	28.70	41.70	60.50	31.80
70-90 lag days	52.39	26.98	33.40	46.77	64.07	30.67
$PM_{2.5-10}$						
0-90 lag days	28.34	15.29	19.31	25.09	34.15	14.84
0-9 lag days	28.31	19.92	16.30	23.98	35.54	19.24
10-14 lag days	28.58	21.52	15.56	23.53	35.61	20.05
70-90 lag days	29.30	18.32	17.86	25.30	36.22	18.36
PM_{10}						
0-90 lag days	77.47	30.74	56.63	72.43	93.17	36.54
0-9 lag days	75.65	38.18	50.00	68.07	92.48	42.48
10-14 lag days	76.32	43.10	47.57	66.89	93.59	46.02
70-90 lag days	91.14	38.23	54.86	72.61	98.63	43.77

0-90 lag days: the entire period of sperm development, 0-9 lag days: the period of epididymal storage, 10-14 lag days: the period of sperm motility development, 70-90 lag days: the periods of spermatogenesis.

© 2022 Zhao Y et al. *JAMA Network Open*.

eTable 2. Mean $PM_{2.5}$, $PM_{2.5–10}$, and PM_{10} Exposure During the Entire Period of Sperm Development (0-90 Days Before Semen Ejaculation) From 2013 to 2019

Years	$PM_{2.5}$ (μg/m^3)			$PM_{2.5-10}$ (μg/m^3)			PM_{10} (μg/m^3)	
	Mean (SD)	Median (P_{25}, P_{75})		Mean (SD)	Median (P_{25}, P_{75})		Mean (SD)	Median (P_{25}, P_{75})
2013	46.9 (19.4)	41.7 (33.1, 55.0)		27.1 (10.9)	23.7 (20.9, 31.2)		69.3 (28.4)	64.0 (54.4, 79.8)
2014	62.8 (22.1)	58.9 (46.3, 74.6)		29.0 (13.8)	26.1 (21.1, 34.5)		89.9 (29.6)	84.9 (70.3, 105.2)
2015	52.4 (17.4)	49.5 (40.1, 64.3)		29.5 (13.7)	26.5 (20.6, 35.7)		81.9 (26.7)	77.8 (62.9, 96.9)
2016	47.8 (19.2)	44.2 (32.5, 60.2)		28.4 (15.3)	24.4 (18.4, 34.4)		76.1 (29.7)	71.6 (53.1, 91.1)
2017	47.4 (21.8)	42.8 (32.8, 56.1)		28.7 (15.8)	25.8 (18.5, 34.9)		76.1 (31.6)	68.2 (55.6, 91.3)
2018	43.0 (18.7)	34.2 (25.6, 48.8)		29.0 (20.8)	21.8 (14.7, 31.1)		72.0 (33.8)	56.9 (44.0, 78.3)
2019	40.3 (20.5)	37.7 (27.2, 51.0)		24.6 (15.0)	23.6 (16.9, 33.3)		64.8 (29.9)	62.4 (47.1, 83.0)

© 2022 Zhao Y et al. *JAMA Network Open*.

eTable 3. Spearman Correlation Coefficients Between PMs and Gaseous Pollutants During the Entire Period of Sperm Development (0-90 Days Before Semen Ejaculation)

	$PM_{2.5-10}$	PM_{10}	SO_2	NO_2	CO	O_3	Temp	RH
$PM_{2.5}$	0.44	0.89	0.64	0.65	0.62	-0.46	-0.67	-0.32
$PM_{2.5-10}$		0.75	0.37	0.22	0.37	-0.14	-0.26	-0.41
PM_{10}			0.60	0.54	0.58	-0.38	-0.58	-0.41
SO_2				0.47	0.55	-0.34	-0.40	-0.30
NO_2					0.48	-0.35	-0.50	-0.26
CO						-0.50	-0.27	-0.18
O_3							-0.63	0.02
Temp								0.28

Temp: temperature, RH: relative humidity

© 2022 Zhao Y et al. *JAMA Network Open*.

eTable 4. Effect Estimates and 95% CIs of Semen Total and Progressive Motility Associated With an IQR Increase in PM Exposure During the Entire Period of Sperm Development (0-90 Days Before Semen Ejaculation) With and Without Adjustment for Smoking and Alcohol Consumption

	**Model 1, %** [a]	**Model 2, %** [b]
**Total motility**		
$M_{2.5}$	-3.60 (-3.93, -3.26) **	-4.41 (-5.00, -3.81) **
$PM_{2.5-10}$	-0.45 (-0.76, -0.14) **	-0.48 (-0.79, -0.17) **
PM_{10}	-2.44 (-2.91, -1.96) **	-2.52 (-3.00, -2.04) **
**Progressive motility**		
$M_{2.5}$	-1.87 (-2.37, -1.36) **	-1.98 (-2.48, -1.47) **
$PM_{2.5-10}$	-0.13 (-0.39, 0.14)	-0.15 (-0.14, 0.12)
PM_{10}	-1.05 (-1.45, -0.64) **	-1.11 (-1.52, -0.71) **

[a] Adjusted for ethnic, age, education, BMI, season of semen collection, abstinence period, temperature, relative humidity and gaseous pollutants, smoking and drinking.

[b] Adjusted for ethnic, age, education, BMI, season of semen collection, abstinence period, temperature, relative humidity and gaseous pollutants.

** $p < 0.01$.

© 2022 Zhao Y et al. *JAMA Network Open*.

eTable 5. Effect Estimates and 95% CIs of Total Motility Associated With an IQR Increase in PM Exposure During the Entire Period of Sperm Development (0-90 Days Before Semen Ejaculation) in Each Subgroup

Subgroups	No. of participants	$PM_{2.5}$, %	$PM_{2.5-10}$, %	PM_{10}, %
**Age, years**				
< 30	8558	-4.46 (-5.67, -3.24) **	-0.56 (-1.23, 0.11)	-3.07 (-4.10, -2.04) **
31-39	20283	-4.01 (-4.76, -3.26) **	-0.15 (-0.54, 0.25)	-1.94 (-2.53, -1.35) **
≥ 40	5035	-5.32 (-6.81, -3.84) **	-1.24 (-1.98, -0.51) **	-3.35 (-4.51, -2.19) **
**BMI, kg/m^2**				
< 24 (underweight or normal)	17691	-4.46 (-5.30, -3.61) **	-0.27 (-0.71, 0.17)	-2.49 (-3.17, -1.81) **
≥ 24 (overweight or obese)	16725	-3.94 (-4.76, -3.12) **	-0.55 (-0.98, -0.12) *	-2.27 (-2.94, -1.61) **
**Education level**				
Middle school or below	4586	-2.46 (-4.14, -0.78) **	-0.07 (-1.07, 0.93)	-1.41 (-2.92, 0.10)
High school	11967	-4.52 (-5.54, -3.51) **	-0.38 (-0.90, 0.14)	-2.36 (-3.18, -1.55) **
College or above	17323	-4.46 (-5.26, -3.66) **	-0.46 (-0.88, -0.04) *	-2.44 (-3.07, -1.82) **

Models adjusted for ethnic, age, education, BMI, smoking, drinking, season of semen collection, abstinence period, temperature, relative humidity, and gaseous pollutants. ** $p < 0.01$, * $p < 0.05$.

© 2022 Zhao Y et al. *JAMA Network Open*.

eTable 6. Effect Estimates and 95% CIs of Progressive Motility Associated With an IQR Increase in PM Exposure During the Entire Period of Sperm Development (0-90 Days Before Semen Ejaculation) in Each Subgroup

Subgroups	No. of participants	$PM_{2.5}$, %	$PM_{2.5-10}$, %	PM_{10}, %
**Age, years**				
< 30	8558	-2.57 (-3.62, -1.52) **	-0.28 (-0.85, 0.30)	-1.69 (-2.58, -0.80) **
31-39	20283	-1.52 (-2.15, -0.88) **	0.17 (-0.15, 0.50)	-0.60 (-1.09, -0.10) *
≥ 40	5035	-2.68 (-3.92, -1.45) **	-0.88 (-1.49, -0.27) **	-1.91 (-2.88, -0.94) **
**BMI, kg/m²**				
< 24 (underweight and normal)	17691	-1.92 (-2.63, -1.20) **	0.02 (-0.35, 0.40)	-1.08 (-1.66, -0.51) **
≥ 24 (overweight and obese)	16725	-1.73 (-2.42, -1.04) **	-0.16 (-0.53, 0.20)	-0.88 (-1.44, -0.32) **
**Education level**				
Middle school or below	4586	-0.98 (-2.41, 0.45)	-0.01 (-0.86, 0.85)	-0.34 (-1.63, 0.94)
High school	11967	-2.00 (-2.87, -1.14) **	0.07 (-0.37, 0.51)	-0.91 (-1.60, -0.21) **
College or above	17323	-1.82 (-2.49, -1.15) **	-0.19 (-0.53, 0.16)	-1.10 (-1.62, -0.58) **

Models adjusted for ethnic, age, education, BMI, smoking, drinking, season of semen collection, abstinence period, temperature, relative humidity, and gaseous pollutants. ** $p < 0.01$, * $p < 0.05$

© 2022 Zhao Y et al. *JAMA Network Open*.

eFigure 1. Location of Participants' Residence Cities

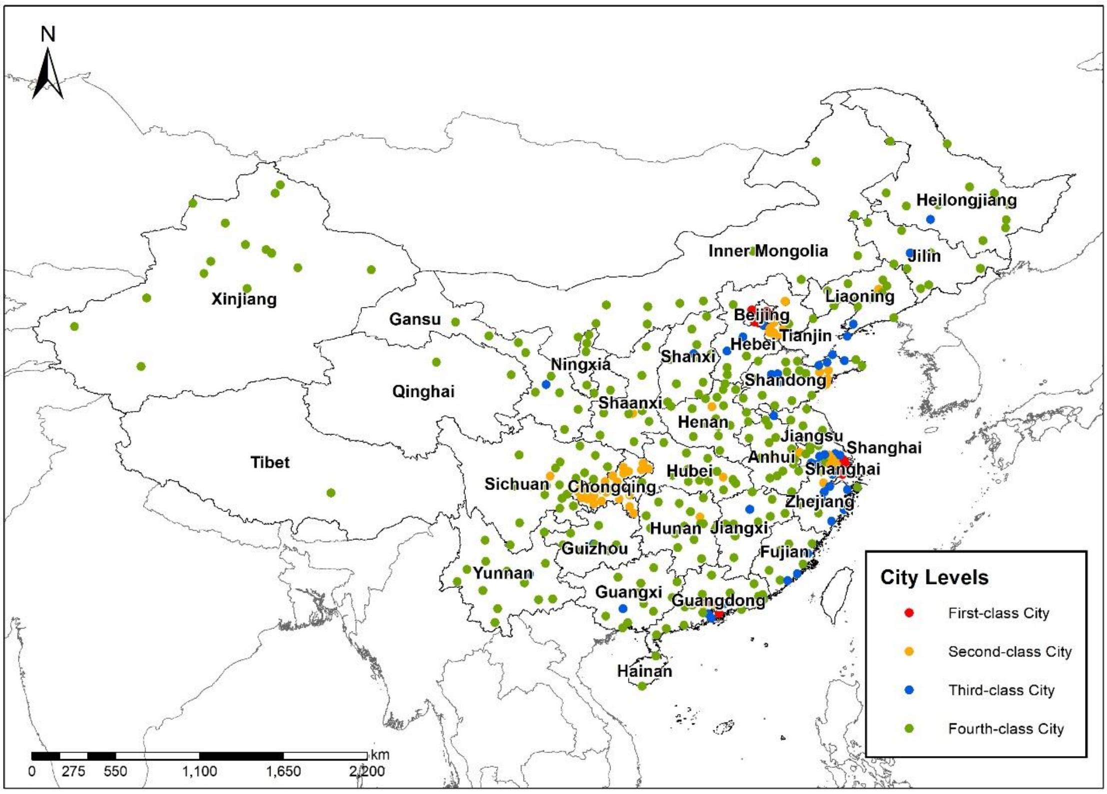

Participants were from 340 prefecture-level cities and resided across the 31 provincial-level administrative regions of China. The participants' residence cities were classified into three classes according to their Gross Domestic Product (GDP) and population of 2019. The red dots represent the first-class cities, which are highly developed mega cities (GDP>2,362 billion RMB and population > 13 million), including Beijing, Shanghai, Guanzhou and Shenzhen. The yellow dots represent the second-class cities included provincial capital cities and developed prefecture-level cities with annual GDP over 500 billion RMB and population over 4 million. And the green dots represent the third-class cities that are all the rest and relatively undeveloped cities.

© 2022 Zhao Y et al. *JAMA Network Open*.

eFigure 2. Distribution of Exposure to $PM_{2.5}$ During the Entire Period Of Sperm Development for Participating Men Residing in Prefecture-Level Cities

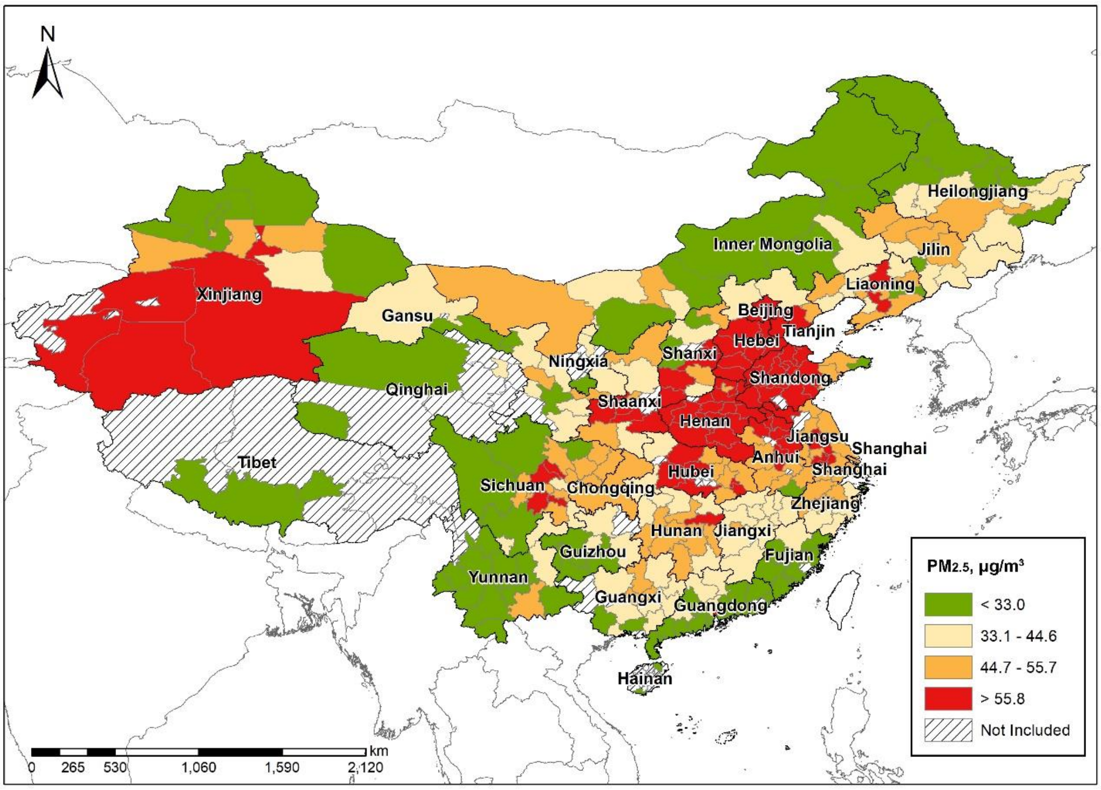

Participants living in the middle and lower reaches of the Yellow River, the Jing-Jin-Ji region and Xinjiang experienced relatively high $PM_{2.5}$ exposure (> 55.80 μg/m^3) over the entire period of sperm development.

© 2022 Zhao Y et al. *JAMA Network Open*.

eFigure 3. Distribution of Exposure to $PM_{2.5\text{-}10}$ During the Entire Period of Sperm Development for Participating Men Residing in Prefecture-Level Cities

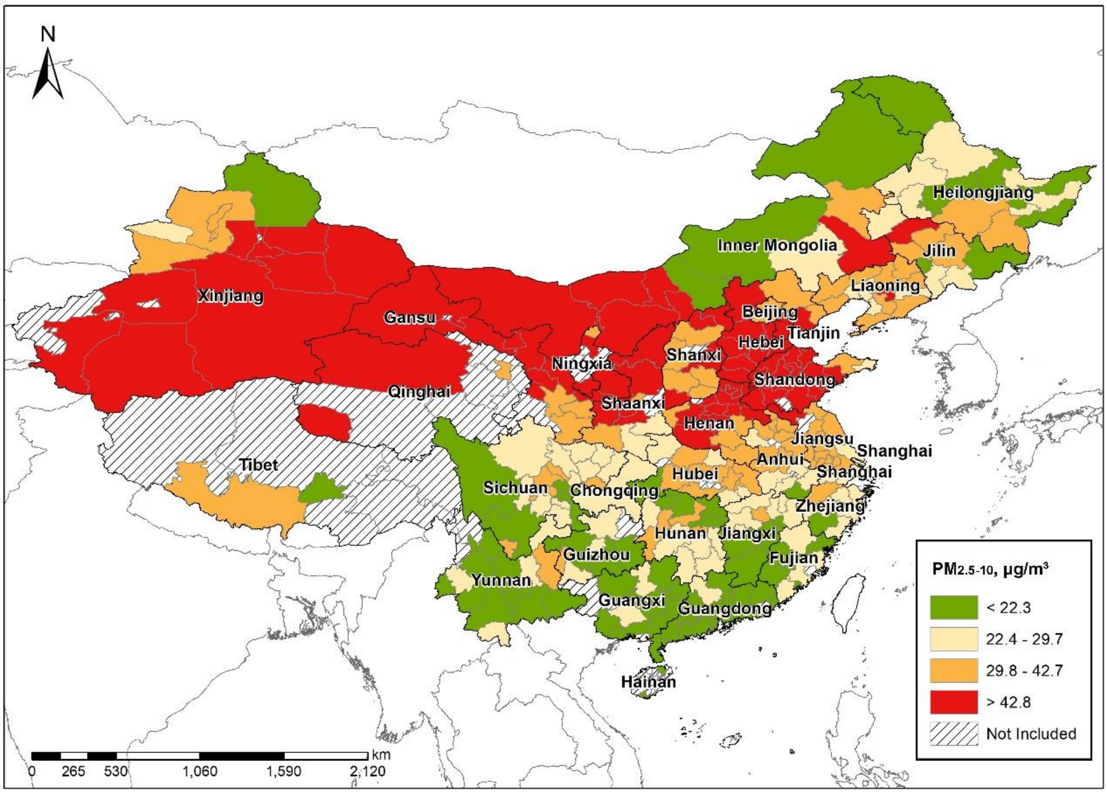

Participants living in the middle and lower reaches of the Yellow River, and the Jing-Jin-Ji region, and Xinjiang, Gansu experienced relatively high $PM_{2.5\text{-}10}$ exposure (> 42.80 μg/m^3) over the entire period of sperm development.

© 2022 Zhao Y et al. *JAMA Network Open*.

eFigure 4. Distribution of Exposure to PM_{10} During the Entire Period of Sperm Development for Participating Men Residing in Prefecture-Level Cities

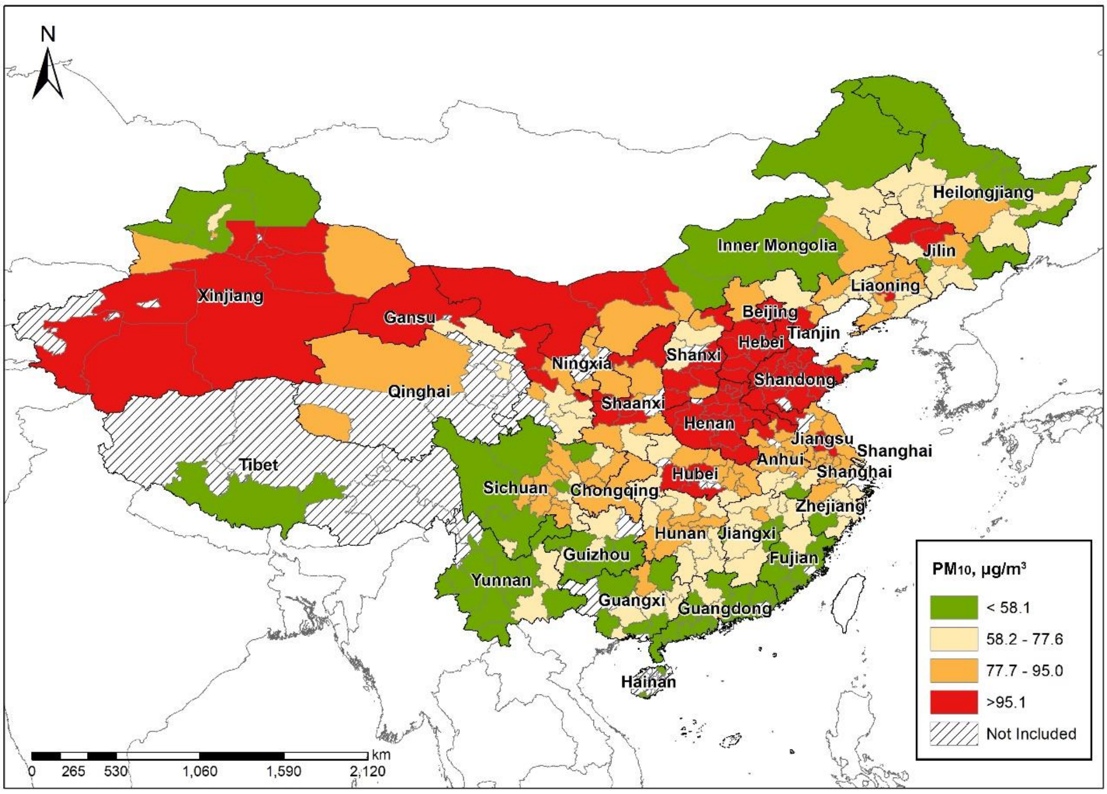

Participants living in the middle and lower reaches of the Yellow River, the Jing-Jin-Ji region and Xinjiang, Gansu experienced relatively high PM_{10} exposure (> 95.10 μg/m^3) over the entire period of sperm development.

© 2022 Zhao Y et al. *JAMA Network Open*.
